# Validation of ACC/AHA and ESC Sudden Cardiac Death Risk Guidelines in Diverse Hypertrophic Cardiomyopathy Cohort: Stratification HCM Study

**DOI:** 10.5334/gh.1380

**Published:** 2024-12-17

**Authors:** Murillo Oliveira Antunes, Fabio Fernandes, Edmundo Arteaga-Fernandez, Félix José Alvarez Ramires, Vinicius Machado Correia, Juliano Novaes Cardoso, Cristhian Espinoza Romero, Henrique Martins Sousa, Marília Taily Soliani, Matheus Ramos Ramos Dal Piaz, Anna Danielle Rodrigues Gandarella, Ruiza Gonçalves Rocha Teixeira, Charles Mady, Caio Assis Moura Tavares, Patricia O. Guimarães, Vagner Madrini Junior

**Affiliations:** 1 Instituto do Coração, Faculdade de Medicina Universidade de São Paulo, São Paulo, Brazil; 2 Universidade de São Francisco, São Paulo, Brazil; 3 Hospital Israelita Albert Einstein, São Paulo, Brazil

**Keywords:** hypertrophic cardiomyopathy, sudden cardiac death, implantable cardioverter-defibrillators

## Abstract

**Background:**

Sudden cardiac death (SCD) is a major concern in patients with hypertrophic cardiomyopathy (HCM). The American College of Cardiology/American Heart Association (ACC/AHA) and the European Society of Cardiology (ESC) have different guidelines for SCD risk stratification. Their comparative performance in diverse populations remains uncertain.

**Objective:**

Evaluate the performance of the 2020 ACC/AHA and 2014 ESC guidelines for SCD stratification in a Brazilian cohort with HCM.

**Methods:**

This retrospective cohort study included patients diagnosed with HCM who were followed in a dedicated clinic at a tertiary hospital in Brazil. The primary outcome was SCD, aborted cardiac arrest due to ventricular fibrillation (VF), sustained ventricular tachycardia (SVT), an episode of VF or SVT, or appropriate ICD therapy. Risk prediction models were assessed using the C-index.

**Results:**

A total of 187 patients were included, with a mean follow-up of 8.3 years. The 2020 ACC/AHA guidelines classified 106 (56%) patients as high-risk for SCD, while the 2014 ESC guidelines identified 54 (29%). The primary outcome occurred in 12% of the high-risk group identified by the ACC/AHA guidelines and 13% of the high-risk group identified by the ESC guidelines. Both guidelines showed low discriminatory power for SCD risk in this Brazilian cohort, with AUC values of 0.634 and 0.581 for the ACC/AHA and ESC guidelines, respectively.

**Conclusions:**

The 2020 ACC/AHA and 2014 ESC guidelines have limitations in predicting SCD events and defining ICD indications in Brazilian HCM patients. Further studies are needed to refine risk stratification and optimize SCD prevention in this population.

## Introduction

Hypertrophic cardiomyopathy (HCM) is a genetic cardiac disorder characterized by unexplained left ventricular hypertrophy (LVH) that cannot be solely explained by loading conditions. HCM is the most common cause of sudden cardiac death (SCD) in young adults and athletes (1). The clinical management of HCM has evolved significantly over the past decades, with a particular emphasis on preventing SCD. Implantable cardioverter-defibrillators (ICDs) have been proven to be effective in preventing SCD in high-risk HCM patients (2).

The American College of Cardiology/American Heart Association (ACC/AHA) and the European Society of Cardiology (ESC) guidelines have different approaches to identify patients at high risk for SCD who may benefit from ICD implantation. The 2020 ACC/AHA guidelines employ a risk-factor approach to recommend ICD implantation, considering five major factors (family history of SCD, unexplained syncope, massive left ventricular hypertrophy [LVH], apical aneurysm, and reduced left ventricular ejection fraction [≤50%]) and two non-major factors (non-sustained ventricular tachycardia [NSVT] and extensive late gadolinium enhancement on cardiac magnetic resonance). The ESC guidelines base their recommendations on a risk-prediction model that estimates the 5-year risk of SCD, considering age, LV wall thickness, left atrial size, maximal left ventricular outflow tract gradient, family history of SCD, NSVT, and the presence of unexplained syncope (3,4), with ICD being recommended for those with estimated SCD risk ≥6% or ≥4% (with different classes of recommendations).

Despite their widespread use, applicability to non-Caucasian populations, such as Brazilians, has not been extensively studied. Brazil is a racially and ethnically diverse country. Thus, it is crucial to validate risk-prediction models in this population to ensure accurate identification of high-risk patients and guide appropriate ICD indications for primary prevention (5,6). Therefore, the aim of this study was to evaluate the diagnostic performance of the 2020 ACC/AHA and 2014 ESC guidelines approaches for SCD risk stratification in a Brazilian cohort with HCM.

## Methods

### Study Population

We retrospectively evaluated 187 patients diagnosed with hypertrophic cardiomyopathy (HCM) at the Heart Institute of São Paulo (INCOR) at the Hospital das Clínicas, Faculty of Medicine, University of São Paulo (HC-FMUSP) between 2012 and 2019. The diagnosis of HCM was defined by echocardiography with a maximum end-diastolic wall thickness ≥15 mm in any left ventricular (LV) segment in the absence of other unexplained causes of ventricular hypertrophy. The following were excluded from the study: patients with hypertension, aortic stenosis and athlete›s heart, primary valve disease, reduced ejection fraction (<50%), phenocopies (e.g., Noonan syndrome, amyloidosis, Fabry disease), under 18 years of age, a follow-up period of less than 5 years, previously subjected to septal reduction therapy (surgical myectomy or septal alcohol ablation), who underwent heart transplantation, ICD for secondary prevention of sudden death, and who were lost to follow-up during the period. Clinical follow-up was conducted through in-person medical appointments or telephone contact for patients who did not attend the medical return. The study was approved by the institution ethics committee.

### Clinical, ECG monitoring, and Imaging parameters

As part of routine care in our institution, all patients underwent 24-hour Holter monitoring, performed at least once a year during the follow-up period. Ventricular tachycardia (VT), both sustained and NSVT, was diagnosed based on electrocardiographic records or 24-hour Holter data, as well as documented arrhythmia histories. NSVT was characterized by the occurrence of three or more consecutive ventricular beats with a frequency ≥100 beats per minute, lasting less than 30 seconds. A positive family history for SCD was defined by the occurrence of unexpected death of a first-degree relative under 50 years of age. Maximum wall thickness was measured by echocardiography, considering the largest measurement obtained in any segment of the LV. Left atrium (LA) and LV dimensions were evaluated following current recommendations. LV ejection fraction was calculated using the biplane Simpson method. The LV outflow tract gradient was measured as described in previous studies. Apical aneurysms were diagnosed by echocardiography or confirmed by cardiac magnetic resonance imaging when necessary. All echocardiographic assessment measurements followed the recommendations of the Cardiovascular Imaging Department of the Brazilian Society of Cardiology.

### Study Outcomes

The primary outcome comprised the occurrence of one of the following findings: SCD, resuscitated cardiac arrest due to ventricular fibrillation (VF) or sustained ventricular tachycardia (SVT), an episode of VF or SVT, or ICD therapy. SCD was defined as a sudden and unexpected collapse occurring within 1 hour of the onset of symptoms in clinically stable patients, resulting in death within 24 hours of presentation. SVT was defined as a ventricular tachycardia faster than 100 beats per minute lasting at least 30 seconds or requiring intervention. For events occurring outside our institution, the circumstances of death were determined by telephone interviews with a family member or based on medical reports. Any disagreement in event information was discussed with three cardiologists and decided by consensus.

### Risk Stratification Algorithms and Outcomes

Patients were assessed for SCD risk according to the 2020 ACC/AHA and 2014 ESC guidelines. The 2020 ACC/AHA risk stratification considers a high risk and a class IIa indication for ICD in the presence of at least one of the five main risk factors: positive family history for SCD, recent unexplained syncope, maximum wall thickness ≥30 mm, LV ejection fraction ≤50%, and the presence of an LV apical aneurysm. The 2014 ESC risk score was also calculated for each study participant using the HCM Risk-SCD model.

All variables used to classify participants according to the 2020 ACC/AHA and 2014 ESC guidelines were obtained at baseline, with missing variables assumed to be absent. At baseline, patients were classified into high- and low-risk groups according to both the ACC/AHA and ESC guidelines. For the ESC guidelines classification, both the 4% and 6% thresholds were used (5-year SCD risk ≥6% [class IIa for ICD] and 5-year SCD risk ≥4% and <6% [class IIb for ICD]).

### Statistical Analysis

Continuous variables are presented as mean and standard deviation or as median and interquartile ranges, as appropriate. Discrete variables are presented as counts and percentages. The student’s t-test was used to compare continuous variables, while the chi-square test was used for categorical variables. The initiation of follow-up was defined as the date of initial evaluation. All participants were followed until the occurrence of death or their most recent outpatient evaluation date, whichever occurred first. Administrative censoring was performed in July 2024. ROC curve analysis was performed to evaluate the risk stratification performance of the 2020 AHA and 2014 ESC proposed SCD risk stratification algorithms.

Kaplan-Meier curves were used to display event-free survival among comparison groups for each guideline. To calculate sensitivity and specificity for each risk stratification algorithm, true positives were defined as individuals who would have received an ICD recommendation according to the algorithm and experienced an SCD event, true negatives as patients who would not have received an ICD recommendation and did not experience an SCD event, false positives as patients who would have received an ICD recommendation without experiencing an SCD event during follow-up, and false negatives as patients who would not have received an ICD recommendation but experienced an MSC event. Statistical significance was defined as a two-tailed P-value < 0.05. All statistical analyses were performed using SPSS version 22.0 (SPSS, Chicago, IL) for Windows.

## Results

### Characteristics of the Study Cohort

Among the 187 patients included in the study, mean age was 41.5 (SD, 14.0) years, and 56% were male. The median follow-up period was 8.3 (IQR 7.4 to 9.1) years. During the follow-up period, 16 events (8.5%) of the primary outcome occurred (individual components: 7 ICD shock, 7 SCDs, 1 NSVT on Holter, and 1 aborted cardiac arrest) (Graphical Abstract). There were no statistically significant differences in clinical or demographic characteristics, echocardiographic findings, or medication use between groups with and without the primary outcome. The obstructive form of HCM was defined as a left ventricular outflow tract (LVOT) gradient exceeding 30 mmHg. Among the participants, 57 individuals (26%) presented with the obstructive form of HCM. Most symptomatic patients are in New York Heart Association (NYHA) functional Class I (53.8%), followed by Class II (36.4%), with a smaller proportion in Class III (9.8%). The majority of patients were treated with beta-blockers (74.9%) and/or amiodarone (22.5%). No patient had a left ventricular ejection fraction below 50% or an apical left ventricular aneurysm. The baseline characteristics are summarized in Table 1.

**Table 1:** Baseline Characteristics of 187 Patients with Hypertrophic Cardiomyopathy (HCM).


CHARACTERISTICS	TOTAL (n = 187)	PRIMARY OUTCOME	p-VALUE

Age, mean ± SD	41.5 ± 14.0	41.6	0.793

Male sex, n (%)	105 (56.1%)	10 (62%)	0.187

Follow-up (years), median (IQR)	8.38 [7.41; 9.1]	7.2	0.187

**Echocardiogram (mm)**			

Septum, median (IQR)	23.8 [20.0; 27.0]	23.8	0.984

Left atrium, median (IQR)	42.9 [38.0; 47.0]	41.7	0.406

Left ventricle, mm	43.2 ± 5.51	41.8	0.293

Ejection fraction (%), mean ± SD	71.5 ± 8.58	70.5	0.615

Obstructive form, n (%)	57 (26%)	5 (31%)	0.572

**SCD Risk Factors**			

Syncope, n (%)	44 (23%)	8 (50%)	0.026

NSVT on Holter, n (%)	58 (31%)	4 (25%)	0.771

Family history of SCD, n (%)	68 (36%)	8 (50%)	0.280

Septum ≥ 30 mm, n (%)	65 (35%)	7 (43%)	0.425

**ACC/AHA 2020**			

High Risk, n (%)	106 (56%)	13 (81%)	0.062

**ESC 2014**			

HCM Risk-SCD ≥ 4%, n (%)	89 (47%)	10 (62%)	0.296

HCM Risk-SCD ≥ 6%, n (%)	54 (29%)	7 (44%)	0.246

**NYHA class**			

I	100 (53,8%)	5 (31%)	0.066

II	68 (36,4%)	7 (41,2%)	0.059

III	19 (9,8%)	4 (27,8%)	0.035

**Medication**			

Beta-blocker, n (%)	140 (75%)	14 (87%)	0.366

Ca+ Channel Blocker, n (%)	44 (23%)	3 (19%)	0.767

Amiodarone, n (%)	42 (22%)	5 (31%)	0.361


Abbreviations: SD, standard deviation; IQR, interquartile range; SCD, sudden cardiac death; NSVT, non-sustained ventricular tachycardia; NYHA, New York Heart Association; ACC/AHA, American College of Cardiology/American Heart Association; ESC, European Society of Cardiology; HCM, Hypertrophic Cardiomyopathy; Ca+, calcium.

Approximately one-third of the population had at least one risk factor for SCD, such as (NSVT) on Holter monitoring, family history of SCD, and interventricular septal thickness (IVS) ≥ 30 mm. Overall, 106 (56%) patients were classified as high risk for SCD (Class IIa ICD indication) by the 2020 ACC/AHA guideline, 89 (47.6%) patients had the HCMRisk-SCD ≥ 4% (Class IIb indication for ICD), and 54 (28.9%) had the HCMRisk-SCD ≥ 6% risk (Class IIa indication for ICD) according to the 2014 ESC guidelines.

### Study Outcomes

Analyzing primary outcome rates in relation to guideline predictions, we observed that out of 106 patients classified as high risk by the American guideline, 13 patients (12%) experienced the primary outcome. Regarding the European guideline, among the 54 patients classified with ≥ 6% SCD risk, 7 patients (13%) experienced the primary outcome. For those 89 patients classified with ≥ 4% and < 6% risk, 10 patients (11%) experienced the primary outcome (Table 2).

**Table 2:** Comparison of Odds Ratio and Number Needed to Treat between Event Prediction Models.


MODEL	HIGH RISK (CDI INDICATED)	LOW RISK (CDI NOT INDICATED)	OR (95% CI)	p-VALUE	NNT (N)

ACC/AHA 2020	13/106	3/81	3.63 (1.0–13.2)	0.06	12

ESC 2014 ≥ 4%	10/89	6/98	1.94 (0.67–5.57)	0.29	16

ESC 2014 ≥ 6%	7/54	9/133	2.05 (0.72–5.82)	0.24	19


Abbreviations: CDI, cardioverter-defibrillator implantation; OR, odds ratio; NNT, number needed to treat.

### Predictors of Sudden Cardiac Death Events

**Figure 1:**
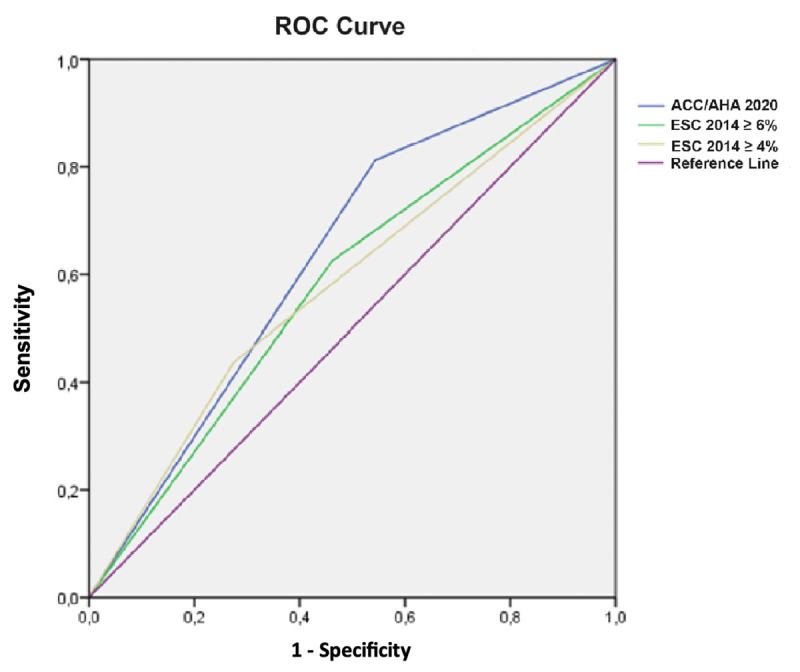
ROC Curves Comparing ACC/AHA 2020 and ESC 2014 Risk Prediction Models. ROC Curves for Risk Prediction Models of ACC/AHA 2020 and ESC 2014 with 4% and 6% Cut-offs. The Sudden Cardiac Death Risk Stratification in ACC/AHA 2020 Guideline Demonstrates a Higher Area under the Curve (0.63) Compared to ESC 2014 Models. ROC: Receiver operating characteristic, ACC/AHA: American College of Cardiology/American Heart Association, ESC: European Society of Cardiology.

The Figure 1 presents ROC curves for the 2020 ACC/AHA guideline criteria and the 2014 ESC guideline at cutoff levels of 4% and 6%: The AUC calculated using the 2020 ACC/AHA guideline was 0.63 (95% CI 0.50–0.76, p = 0.05), with sensitivity and specificity of 81% and 46%, respectively (Table 3).

**Table 3:** Comparison of Sensitivity, Specificity, and Accuracy between Event Prediction Models.


MODEL	AUC (95% CI)	SENSITIVITY (%) (95% CI)	SPECIFICITY (%) (95% CI)	NPV (%) (95% CI)	ACCURACY (%) (95% CI)

ACC/AHA 2020	0.63	81 (62.1–99.9)	45 (38.1–53.1)	96 (92.2–99.9)	49 (41.5–55.8)

ESC 2014 ≥ 4%	0.58	62 (38.8–86.2)	54 (46.3–61.3)	94 (89.1–98.6)	54 (47.4–61.7)

ESC 2014 ≥ 6%	0.58	44 (19.4–68.1)	72 (65.8–79.2)	93 (89.0–97.5)	54 (63.5–76.6)


Abbreviations: AUC, area under the curve; CI, confidence interval; NPV, negative predictive value.

For the ESC 2014 model with a 4% cutoff, the AUC was 0.58 (95% CI 0.43–0.72, p = 0.17), with sensitivity and specificity of 65% and 54%, respectively. At a 6% cutoff, the AUC was 0.58 (95% CI 0.42–0.73, p = 0.17), with sensitivity and specificity of 44% and 72%, respectively (Table 3).

**Figure 2:**
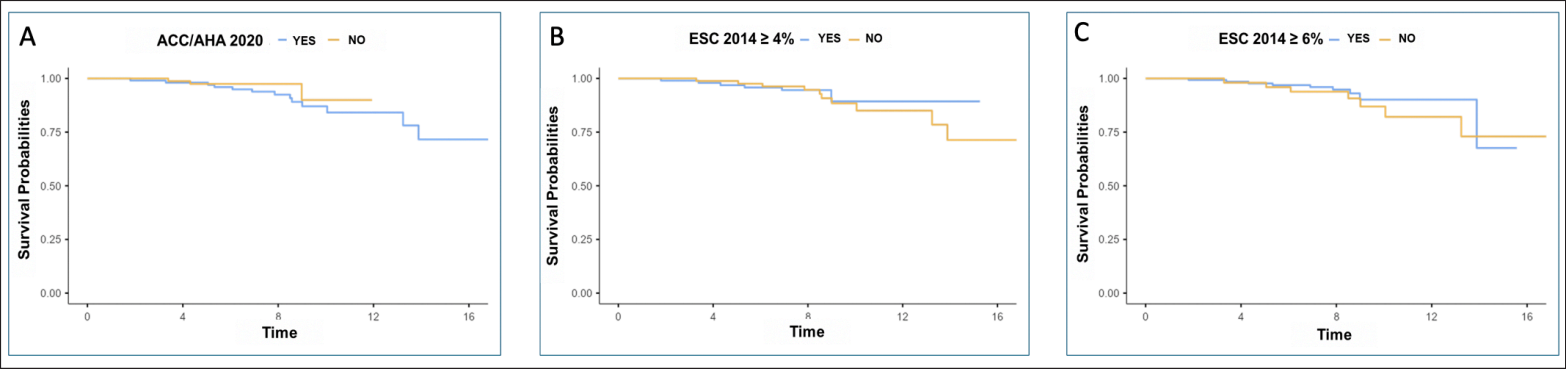
Kaplan-Meier Curves for Survival Probabilities by Risk Stratification Models. Kaplan-Meier Curves for Overall Survival Free from All-Cause Mortality by Risk Stratifications Based on ACC/AHA 2020 **(A)**, ESC 2014 Guideline with 4% Cut-off **(B)**, and 6% Cut-off **(C)**. ACC/AHA: American College of Cardiology/American Heart Association, ESC: European Society of Cardiology.

Survival analysis using Kaplan-Meier curves for event-free survival from SCD compared high- and low-risk groups based on ACC/AHA scores and 4% and 6% ESC cutoffs. No statistically significant differences were observed between groups in all tested categories (Figure 2).

In order to compare the predictive ability of SCD risk stratifications recommended by two2020 ACC/AHA and 2014 ESC guidelines, the odds ratio (OR) and number needed to treat (NNT) to prevent one case of SCD are presented in Table 3. The results indicate that according to the 2020 ACC/AHA guideline, 12 ICD implants are needed to prevent one case of SCD over 5 years. Using the 2014 ESC model with a 4% cutoff, 16 ICD implants are needed to prevent one case of SCD. Using the cutoff > 6% for the 2014 ESC model, 19 ICD implants are needed to prevent one case of SCD, indicating less efficacy in identifying patients at high risk for SCD.

## Discussion

This study represents the first comparative evaluation of SCD risk stratification methods recommended by the 2020 ACC/AHA and 2014 ESC guidelines in a Brazilian cohort of patients with HCM. Our findings underscore the complexity and challenges associated with SCD risk stratification in HCM patients. While both the ACC/AHA and ESC guidelines propose specific algorithms to predict SCD risk and guide ICD indications in a cost-effective manner, our analysis reveals limited efficacy of these tools in the Brazilian population (3).

In our results, application of the ACC/AHA 2020 guideline for primary prevention ICD indication showed higher sensitivity and negative predictive value but lower specificity compared to the ESC 2014 score. The American guideline correctly identified 81% of primary events in our sample, whereas the European guideline correctly identified 62% and 43% of events for the 4% and 6% risk strata, respectively. ROC curve analysis revealed that both ESC and ACC/AHA risk scores had areas under the curve below expected values, indicating questionable accuracy in predicting events for ICD indication (2,8).

This finding suggests that a substantial portion of our sample underwent ICD implantation without translating into real patient benefit, considering associated complications and costs. Conversely, while the 2014 ESC model showed higher specificity, it failed to identify all patients who experienced primary events, raising concerns about the sensitivity of these guidelines in preventing SCD (3,7). Although our findings align with those reported by other authors, the values observed in our population are lower compared to previous studies. For instance, Zerkos et al. (8,9) reported a sensitivity of 96% for the ACC/AHA 2020 score in a large cohort of HCM patients.

Another significant finding in our cohort was the high prevalence of SCD risk factors. However, the presence of these factors did not directly correlate with the occurrence of primary events, suggesting that in HCM—a genetically heterogeneous disease—other yet unknown factors may influence SCD risk (10). No significant differences between high- and low-risk groups defined by risk scores were observed in the survival analysis. This indicates that while these scores provide a framework for clinical decision-making, they may not fully capture the heterogeneity and complexity of HCM patients. Our results underscore the need for additional studies to validate and possibly refine these prediction models, considering the specific characteristics of our population (11,12).

## Limitations

Limitations need to be acknowledged. First, this is a single-center study, which limits the generalizability of the results to other populations and settings. Second, the small sample size may increase the risk of type I error. However, the long follow-up period provides individual patient validation and real-world implications of using different risk scoring systems. Third, the low event rate during follow-up reduces the capability of evaluating all the outcomes individually. Fourth, the unavailability of echocardiographic data on systolic anterior motion (SAM) of the mitral valve for all patients may impact the assessment of left ventricular outflow tract gradients. Additionally, since rhythm documentation was not available for all SCD cases, some of these deaths may have been non-arrhythmic in nature. Furthermore, cardiac magnetic resonance (CMR) was not performed in all patients, limiting our ability to assess the fibrosis profile, as recommended in ACC/AHA guidelines.

## Conclusion

Guidelines for ICD indication from major cardiology societies, validated in American and European populations such as the 2020 ACC/AHA and 2014 ESC, demonstrate significant limitations when applied to Brazilian patients. Given that these results are derived from a sample localized in one Brazilian center, caution should be taken in generalizing them to other populations. Future studies should focus on validating these models within our population, utilizing individualized approaches and exploring new strategies to enhance accuracy in risk prediction and therapeutic decision-making for HCM patients in Brazil.

The findings of this study underscore the need for a tailored approach when applying SCD risk stratification guidelines for HCM patients in ethnically and genetically diverse populations. Given the limited predictive accuracy of the 2020 ACC/AHA and 2014 ESC guidelines in the Brazilian HCM cohort, clinicians should consider individual patient characteristics when recommending ICD implantation. This highlights the importance of advancing medical knowledge and procedural skills, emphasizing continuous practice-based learning and the refinement of risk prediction models to enhance clinical decision-making. Furthermore, it points to the necessity of future research to validate and adapt these models for genetic and phenotypic diversity, fostering collaborative efforts to develop more accurate, culturally relevant tools for improving HCM management in Brazil and other diverse populations.

## Graphical Abstract

**Graphical Abstract:**
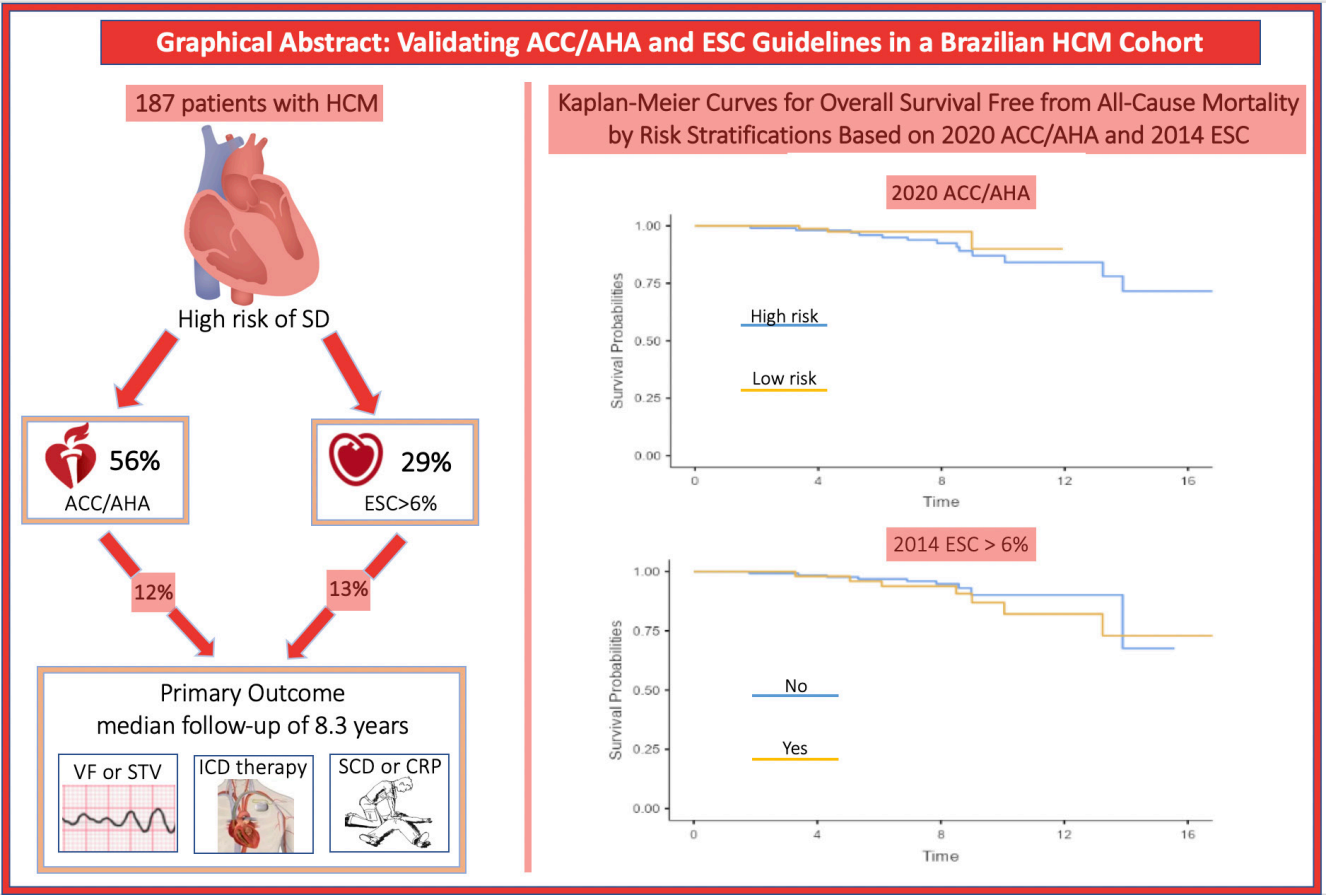
Comparative Validation of ACC/AHA and ESC Guidelines for Sudden Death Risk Stratification in a Brazilian Hypertrophic Cardiomyopathy Cohort. ACC/AHA and ESC guidelines for sudden death (SD) risk in 187 Brazilian hypertrophic cardiomyopathy (HCM) patients. The ACC/AHA guidelines identified 56% as high risk, while the ESC guidelines identified 29% (threshold >6%). Over 8.3 years, primary outcomes (VF, STV, ICD therapy, SCD/CRP) had event rates of 12% (ACC/AHA) and 13% (ESC). Kaplan-Meier curves show overall survival, comparing high- and low-risk groups, illustrating the guidelines’ effectiveness in predicting outcomes. ACC/AHA: American College of Cardiology/American Heart Association, ESC: European Society of Cardiology, HCM: Hypertrophic Cardiomyopathy, SD: Sudden Death, VF: Ventricular Fibrillation, STV: Sustained Ventricular Tachycardia, ICD: Implantable Cardioverter-Defibrillator, SCD: Sudden Cardiac Death, CRP: Cardiac-Related Procedures.

## Abbreviations

HCM – Hypertrophic cardiomyopathy

LVH – Left ventricular hypertrophy

SCD – Sudden cardiac death

ICD – Implantable cardioverter-defibrillator

ACC – American College of Cardiology

AHA – American Heart Association

ESC – European Society of Cardiology

NSVT – Non-sustained ventricular tachycardia

LA – Left atrium

LV – Left ventricle

VT – Ventricular tachycardia

VF – Ventricular fibrillation

SVT – Sustained ventricular tachycardia

INCOR – Heart Institute of São Paulo

HC-FMUSP – Hospital das Clínicas, Faculty of Medicine, University of São Paulo

IVS – Interventricular septal thickness

NNT – Number needed to treat

ROC – Receiver operating characteristic

AUC – Area under the curve

SPSS – Statistical Package for the Social Sciences
